# Prehabilitation to Improve Outcomes of Patients with Gynaecological Cancer: A New Window of Opportunity?

**DOI:** 10.3390/cancers14143448

**Published:** 2022-07-15

**Authors:** Joëlle Dhanis, Nathaniel Keidan, Dominic Blake, Stuart Rundle, Dieuwke Strijker, Maaike van Ham, Johanna M. A. Pijnenborg, Anke Smits

**Affiliations:** 1 Department of Obstetrics and Gynaecology, Radboud University Medical Centre, 6525 GA Nijmegen, The Netherlands; maaike.vanham@radboudumc.nl (M.v.H.); hanny.ma.pijnenborg@radboudumc.nl (J.M.A.P.); anke.smits@radboudumc.nl (A.S.); 2 Faculty of Medical Sciences, Radboud University, 6526 GA Nijmegen, The Netherlands; 3 Department of Gynaecological Oncology, Queen Elizabeth Hospital, Gateshead NE9 6SX, UK; nathaniel.keidan2@northumbria-healthcare.nhs.uk (N.K.); dominic.blake@nhs.net (D.B.); stuart.rundle@nhs.net (S.R.); 4 Department of Surgery, Radboud University Medical Centre, 6525 GA Nijmegen, The Netherlands; dieuwke.strijker@radboudumc.nl

**Keywords:** prehabilitation, gynaecological cancer, endometrial cancer, ovarian cancer, cervical cancer, quality of life, operative complications

## Abstract

**Simple Summary:**

Surgery is an important mainstay in the treatment of gynaecological cancers but is associated with operative complications, especially for those with poor physical and mental health. Prehabilitation is a new and upcoming strategy to optimise patients’ functional capacity, nutritional status and psychosocial wellbeing in order to reduce complications and enhance recovery. In this review, we assessed the evidence on prehabilitation programmes for patients with gynaecological cancer. The limited evidence shows that prehabilitation may reduce length of hospital stay for ovarian cancer patients, and may result in significant weight loss in patients with endometrial and cervical cancer. Comparative prospective studies are required to determine the effectiveness of prehabilitation on reducing operative complications and improving quality of life, and to further specify the content of such a programme for patients with gynaecological cancer.

**Abstract:**

The literature evaluating the effect of prehabilitation programmes on postoperative outcomes and quality of life of patients with gynaecological cancer undergoing surgery was reviewed. Databases including Pubmed, Medline, EMBASE (Ovid) and PsycINFO were systematically searched to identify studies evaluating the effect of prehabilitation programmes on patients with gynaecological cancer. Both unimodal and multimodal prehabilitation programmes were included encompassing physical exercise and nutritional and psychological support. Primary outcomes were surgical complications and quality of life. Secondary outcomes were anthropometric changes and adherence to the prehabilitation programme. Seven studies fulfilled the inclusion criteria, comprising 580 patients. Included studies were nonrandomised prospective studies (n = 4), retrospective studies (n = 2) and one case report. Unimodal programmes and multimodal programmes were included. In patients with ovarian cancer, multimodal prehabilitation resulted in significantly reduced hospital stay and time to chemotherapy. In patients with endometrial and cervical cancer, prehabilitation was associated with significant weight loss, but had no significant effects on surgical complications or mortality. No adverse events of the programmes were reported. Evidence on the effect of prehabilitation for patients with gynaecological cancer is limited. Future studies are needed to determine the effects on postoperative complications and quality of life.

## 1. Introduction

Gynaecological malignancies account for approximately 13% of all new cancer diagnoses in women in Europe [1]. Surgery is an important mainstay of treatment for these women. However, surgery is associated with significant risks on both intraoperative (4.7%) and postoperative complications (25.9%) [2]. The majority of the gynaecological cancer population, especially endometrial and vulvar cancer patients, are characterised by advanced age, obesity and a sedentary lifestyle. Endometrial cancer has the strongest association with obesity among all cancers in women. Ovarian cancer patients are further characterised by malnourishment due to tumour growth and decreased intake [3,4,5,6,7,8,9]. Consequently, gynaecological cancer patients are often high-risk surgical patients.

Surgical complications significantly impede recovery and negatively impact quality of life of patients. Furthermore, they may delay adjuvant therapies, with a subsequent detrimental effect on survival [10,11,12]. Current approaches to reduce surgical complications have focused mainly on postoperative care. The widely adopted Enhanced Recovery After Surgery (ERAS) protocol has been shown to significantly reduce surgical complication rates and hospital stay [13,14,15]. However, this approach fails to address modifiable preoperative risk factors such as physical fitness, nutritional status and smoking. Prehabilitation programmes have been shown to improve operative outcomes and quality of life through optimisation of patients prior to surgery [16]. Several prehabilitation programmes are well established for orthopaedic and cardiac surgery and have recently been introduced in colorectal and abdominal cancer surgery [17,18,19,20,21].

Following similarities in risk factors targeted by prehabilitation among different cancer sites, prehabilitation has the potential to improve outcomes for patients with gynaecological cancer. A systematic search and narrative review were therefore performed to assess the effectiveness of prehabilitation programmes on surgical complications, mortality and quality of life of gynaecological cancer patients undergoing surgical treatment.

## 2. Materials and Methods

### 2.1. Criteria for Considering Studies for This Review

This review was performed according to Preferred Reporting Items for Systematic reviews and Meta-Analyses (PRISMA) guidelines [22]. Studies evaluating the effectiveness of prehabilitation programmes on postoperative and quality of life outcomes in patients with gynaecological cancer were identified. Both unimodal and multimodal interventions consisting of physical exercise, nutritional support, smoking cessation, reduction in alcohol consumption or psychological support were included. Eligible study designs included randomised controlled trials (RCTs), cohort studies, pilot studies, feasibility studies and case reports. Studies were excluded if there was no full-text availability or they were not available in the English language. In addition, studies evaluating a prehabilitation programme with a duration of less than 3 days were excluded. Reviews were assessed for eligible studies. Primary outcomes were defined as surgical complications and mortality. Complications included intraoperative complications such as injuries, blood loss and conversion rates, and postoperative complications including infection, pain, haemorrhage, wound problems, pulmonary complications, venous-thromboembolism and hospital stay. Secondary outcomes were defined as time from surgery to starting adjuvant treatment, quality of life and anthropometric changes.

### 2.2. Search Strategy

The search protocol was based on the PRISMA guidelines [22]. The protocol was not registered prior to performing the search. A comprehensive search of studies evaluating the effectiveness of a prehabilitation programme for gynaecological cancer patients was performed. Pubmed, Medline, EMBASE (Ovid) and PsycINFO databases were systematically searched. The complete search strategy included keywords and MeSH terms related to the review topic (Appendix A). For current and upcoming clinical trials, ClinicalTrials.gov was searched. In addition, the reference lists of eligible studies were assessed to identify additional studies for inclusion. The search was performed in December 2021 and assessed studies published between 1972 and 2021.

### 2.3. Study Selection, Data Extraction and Analysis

Two reviewers (J.D. and N.K) independently assessed publication titles and abstracts of all identified studies according to the inclusion criteria. Potentially relevant studies were retrieved in full text and further assessed for eligibility by both reviewers. Any disparities were resolved by discussion with a third independent reviewer (A.S.). The following variables were extracted by both reviewers: type of study, country, year of publication, population, sort cancer and stage, type of prehabilitation programme, measures, time points and outcomes.

### 2.4. Assessment of Risk of Bias

Study bias was assessed using the Risk Of Bias In Non-Randomised Studies of Interventions (ROBINS-I) presented by the Cochrane Collaboration for nonrandomised cohort studies [23]. This assessment tool assesses bias due to confounding, participant selection, classification of interventions, deviations from intended interventions, missing data, outcome measurements and the selection of reported results. The Joanna Briggs Institute Critical Appraisal Checklist for Case Reports was used to assess bias in case reports [24]. This tool evaluates the presence of demographic characteristics, patients’ history, clinical condition, diagnostic tests and results, interventions, postintervention clinical condition, adverse events and takeaway lessons. Three reviewers (J.D., N.K. and A.S.) assessed bias and resolved differences by discussion.

## 3. Results

### 3.1. Study Selection

**Figure 1:**
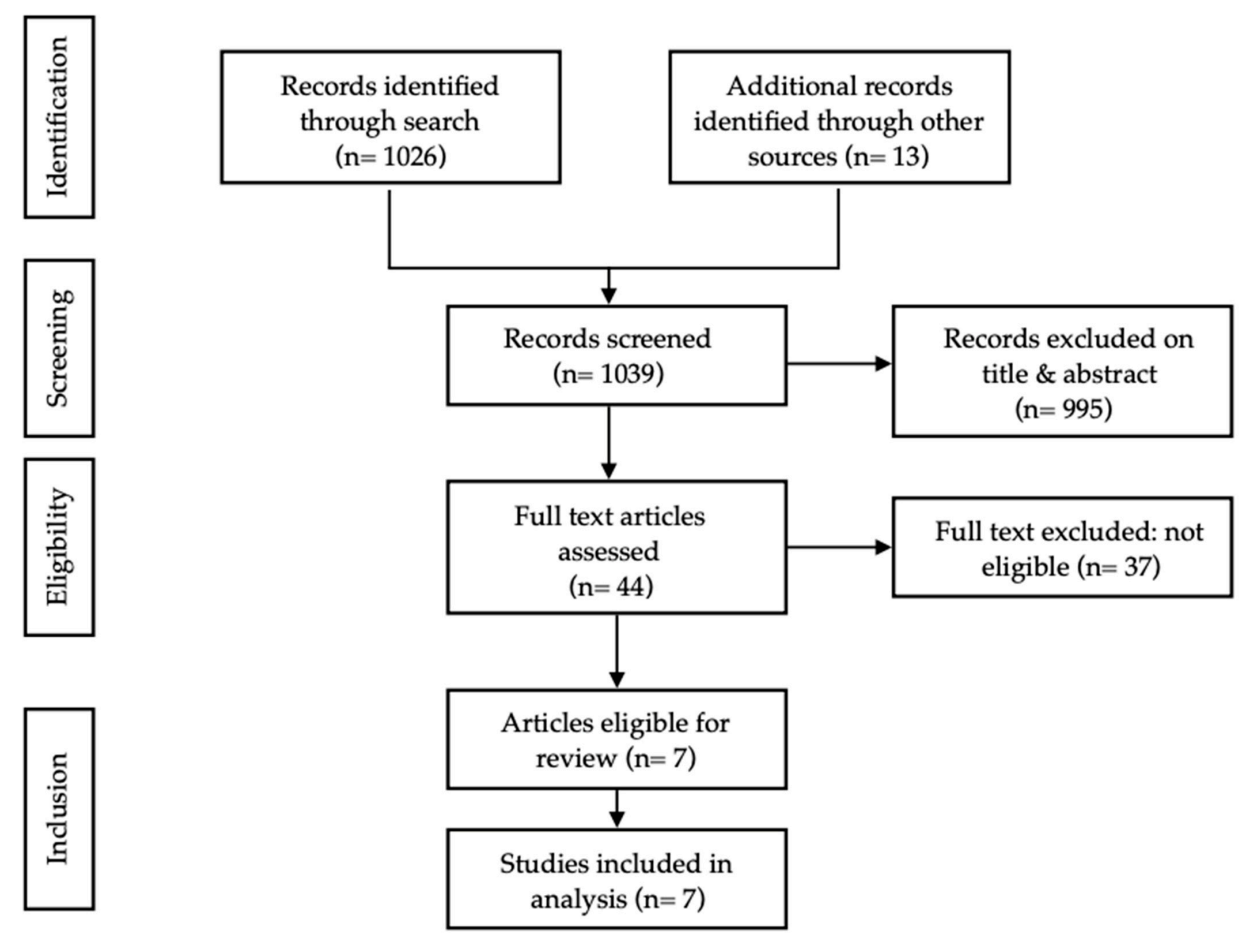
Flow diagram selection of studies.

A total of 1026 articles were identified. Following title and abstract review, 31 articles were retrieved in full text, of which five met the inclusion criteria. A search of reference lists identified a further 13 articles, of which two were eligible for inclusion. This resulted in the inclusion of seven unique articles (Figure 1). Studies were grouped according to type of cancer included.

### 3.2. Included Studies

Four of the included studies were nonrandomised prospective studies [25,26,27,28], two were retrospective studies [29,30] and one was a case report [31]. All studies were single-institution designs, with five studies being comparative studies, comparing prehabilitation to standard care [25,26,27,28,30]. The study by Diaz-Feijoo et al. was included as a preliminary report prior to their later publication in 2022 [28,32]. Five hundred eighty patients with gynaecological cancer were included in the studies, of which 212 participated in a prehabilitation programme. The number of patients per study ranged from 34 to 294 patients. Characteristics of included studies are illustrated per cancer group in Table 1 (ovarian cancer) and Table 2 (endometrial and cervical cancer). Four studies reported predominantly on ovarian cancer patients [26,27,28,30]. Mean age of this population varied between 55 and 70 years [26,28]. BMI was reported in one study, with a median of 24 kg/m^2^, and severity of comorbidities reported was moderate (Charlson comorbidity index 4, ASA 2–3) [26,28]. The three other studies mainly included endometrial or cervical cancer patients [25,29,31]. Mean age of study populations varied between 54 and 58 years [25,29]. Comorbidities reported were hypertension and diabetes, and were prevalent in the majority of patients in the study of Aubrey et al., with a median BMI of 48 kg/m^2^ [29]. Two studies assessed multimodal prehabilitation programmes [27,28], and the remaining studies assessed unimodal programmes [25,26,29,30], with types of interventions shown in Table 1 and Table 2. Three studies evaluate preoperative programmes only, while one study continued the intervention postoperatively [26]. Finally, the case report described an individualised programme for an endometrial cancer patient [31].

### 3.3. Ovarian Cancer

#### Prehabilitation Programmes

Four studies reported on a prehabilitation programme in patients with ovarian cancer, including a total of 428 patients, the vast majority of whom underwent surgery for confirmed or suspected cancer (Table 1) [26,27,28,30]. Two studies reported on multimodal programmes [27,28]. Diaz-Feijoo et al. (intervention group (IG) n = 15, control group (CG) n = 19) assessed a programme consisting of physical therapy, psychological support and nutritional optimisation. Physical therapy consisted of supervised three weekly exercises, consisting of endurance training on a cyclo-ergometer or treadmill and strength (resistance) training. In addition, patients underwent respiratory physiotherapy with a spirometer. Psychological support was delivered through group sessions to reduce anxiety and support appropriate coping. Nutritional optimisation included individualised diets, protein supplements and preoperative immunonutrition. Outcomes were defined as postoperative complications (up to 30 days), length of hospital stay, time until adjuvant chemotherapy and adherence to the programme [28]. Seibaek et al. (IG n = 55, CG n = 90) reported on a programme consisting of physical therapy, psychological support, nutritional supplementation and smoking cessation. Physical therapy consisted of early physiotherapy supporting respiration and circulation, but was not further specified. Psychological support was delivered through individual telephone coaching and the provision of written and visual information. Exact duration of the preoperative programme was not specified in weeks. Outcome measurements included quality of life (Short-Form 36 (SF-36)) and the life orientation (SOC; sense of coherence) questionnaire preoperatively and eight weeks postoperatively. Women with suspected ovarian cancer and ascites were allocated to the intervention, with the control group being women with suspected ovarian cancer without ascites [27]. Two other studies evaluated a unimodal programme of nutritional optimisation with immunonutrition supplements in comparison with a control group with a normal varied diet [26,30]. Fernández-Candela et al. (IG n = 48, CG n = 59) administered immunonutrition (Atempero^R^; containing L-arginine, omega-3 fatty acids and nucleotides) twice daily for seven days preoperatively. The intervention group differed from the control group with more extensive visceral resections, more often chemotherapy and less frequent administration of hyperthermic intraperitoneal chemotherapy (HIPEC). The effect of nutritional support was evaluated through length of hospital stay, postoperative complications, biochemical parameters (serum albumin and CRP), and adherence [30]. Hertlein et al. assessed immunonutrition (Impact^R^; containing arginine, nucleotides and omega-3 fatty acids with a high-calorie formula) given three times daily for five days pre- and postoperatively in malnourished patients (IG n = 28, CG n = 19). Outcome measures included length of stay, postoperative complications and compliance [26].

### 3.4. Effectiveness of Prehabilitation Programmes

Diaz-Feijoo et al. reported a significantly shorter length of stay of 2 days in the intervention group, and also a significantly shorter time until start of chemotherapy (10 days; IG 25 days and CG 35 days). There was no significant difference in major complications, defined as Clavien–Dindo ≥ 3 (respectively, 40% in IG and 63% in CG), and no major adverse events occurred [28]. Seibaek et al. assessed quality of life and showed a significantly higher physical functioning In the control group compared to the intervention group after completion of the programme (mean difference of 10.88 points on physical functioning using Short-Form 36 (SF-36), scale 0–100). Other quality of life domains did not show significant differences between groups. However, groups were not comparable in terms of age and diagnosis, nor was baseline functioning assessed prior to starting the programme. Postoperatively, physical functioning did not retain its statistical difference. A difference in complications between the intervention and control group was not assessed [27]. Fernández-Candela et al. found immunonutrition not to be associated with reduced operative complications, assessed by the Clavien–Dindo classification. However, a multivariate subgroup analysis showed that immunonutrition was associated with a reduction in major complications (Clavien–Dindo III-V) with an odds ratio of 0.247 (CI: 0.071–0.895). No other differences were found between immunonutrition and outcomes [30]. Hertlein et al. did not observe a significant effect of immunonutrition on any of the outcomes assessed, including postoperative complications and length of hospital stay [26]. Adherence to the programme was assessed by two studies. In the study by Diaz-Feijoo et al., overall adherence to the prehabilitation programme was deemed satisfactory in 80% of the participants [28]. Hertlein et al. assessed the compliance of immunonutrition intake pre- and postoperatively and found a satisfactory compliance (defined as 8–11 of 15 servings) of 78.6% preoperatively. Optimal compliance (defined as 12–15 servings) was 60.7% and 21.4%, respectively. Reasons for noncompliance were poor motivation and nausea and vomiting [26].

### 3.5. Endometrial Cancer and Cervical Cancer

#### Prehabilitation Programmes

Two studies reported on a unimodal prehabilitation programme for gynaecological cancer patients (nutrition and psychological therapy). Aubrey et al. assessed mostly patients with endometrial cancer patients (63%) but also included endometrial hyperplasia and patients with adnexal masses [25,29] (Table 2). A study by Arnaboldi et al. included a majority of patients with (recurrent or progressive) cervical (59.2%) and endometrial (14.3%) cancer undergoing pelvic exenteration (Table 2). An additional case report evaluated the role of a multimodal prehabilitation programme, containing physical therapy and nutritional optimisation [31] (Table 2). Aubrey et al. compared three types of weight loss interventions in 48 patients with a BMI > 40 kg/m^2^: a low-calorie liquid diet utilising Optifast 900 for up to 12 weeks; a structured low-calorie meal plan with or without concomitant antiobesity medication (AOM) (GLP-1 analogues or extended-release naltrexone-bupropion); or a six-month programme involving dietician-supported dietary change and AOM, followed by a low-calorie liquid diet. During weight loss interventions, medical management was initiated in 12/30 women (progesterone-containing intrauterine or an aromatase inhibitor) with either grade 1 endometrioid carcinoma or atypical endometrial hyperplasia. Outcome measures included weight loss (%), surgical time, estimated blood loss, conversion rates to laparotomy and hospital stay [29]. The unimodal study by Arnaboldi et al. assessed the introduction of a preoperative psychological intervention on postoperative pain (Visual Analogue Scale; VAS) and length of hospital stay. Gynaecological patients presenting with high levels of psychological distress (Psychological Distress Inventory (PDI) ≥ 30) were offered preoperative telephone counselling and face-to-face interviews throughout their hospital stay. Outcomes were compared to patients with lower levels of psychological distress who did not receive an intervention (PDI < 30) [25]. Finally, Carli et al. described an individualised 3-week home-based programme consisting of physical therapy and nutritional optimisation for an 88-year-old endometrial cancer patient. Physical therapy involved thrice weekly training which included strengthening exercises, abdominal breathing exercises and supervised walking. Nutritional support focused on increasing dietary intake of protein and calories. Outcome measures consisted of exercise tolerance (six-minute walk test), cognitive function (Repeatable Battery for the Assessment of Neuropsychological Status), quality of life (SF-36) and perioperative complications [31].

### 3.6. Effectiveness of Prehabilitation Programmes

Aubrey et al. found that weight loss interventions lead to a significant reduction in weight from initial consultation to end of the intervention (mean weight difference = 12.0 kg, 9.7%). However, they did not specify between the different interventions, with the majority of women receiving the low-calorie liquid diet up to 12 weeks (n = 39). Median number of weight loss clinic visits was 7 days. There was no significant association between BMI, weight loss and surgical outcomes. Arnaboldi et al. evaluated postoperative pain and length of hospitalisation between the intervention (PDI < 30) and control group (PDI ≥ 30). No significant difference in outcomes between the groups was observed. However, comparability of groups, besides PDI scores, was not reported [25]. Finally, the case report by Carli et al. reported that no perioperative complications occurred, and that individual improvements were seen in exercise tolerance, cognitive function and several components of quality of life following the programme [31].

### 3.7. Quality of the Studies

Six of the included studies were nonrandomised cohort studies [25,26,27,28,29,30]. An evaluation of bias using the ROBINS-I tool is illustrated in Table 3 for these studies [23]. All studies were considered to be at moderate risk of bias according to the ROBINS-I tool primarily due to their selection of participants and selection of reported results. In addition, three of the studies were found to be at serious risk of bias due to missing information used to determine which patients were offered interventions [25]; subjective methods used to measure outcomes, such as questionnaires [27]; and significant differences in standard care between intervention and control groups [30]. Several studies had a significant selection bias, with Arnaboldi et al. allocating patients to the intervention based on their preoperative psychological distress inventory (PDI) score and Seibaek et al. allocating patients to the intervention based on presence of ascites [25,27]. Fernández-Candela et al. reported significant differences in baseline characteristics between intervention and control groups [30]. All studies clearly described the prehabilitation interventions administered to patients. The case report was considered as being of low risk of bias, using the Joanna Briggs Institute (JBI) Critical Appraisal Checklist for Case Reports [31].

## 4. Discussion

This review summarises the existing literature on the effectiveness of prehabilitation programmes on postoperative complications and quality of life of patients with gynaecologic cancer. Although prehabilitation is shown to significantly improve postoperative outcomes of patients in other surgical fields, this review demonstrates that evidence of beneficial effects in gynaecological cancer is not completely clear.

A total of seven studies were identified evaluating a prehabilitation programme for gynaecological cancer patients in which considerable heterogeneity of interventions and outcome measures were observed. One study of relatively high methodological quality, with adequate handling of confounders and parity of interventions, reported significant improvements in time to adjuvant treatment and shorter hospital stays in ovarian cancer patients [28]. As chemotherapy is an integral part of ovarian cancer management, a delay in adjuvant treatment beyond 25 to 35 days due to surgical morbidity will result in a significantly poorer prognosis [29]. Other studies, however, failed to demonstrate such improvements [26,27,28,30]. Whilst this may be related to differences in the outcome measures reported, it is also likely that bias related to confounding and selection impacted on the ability of these studies to accurately measure the true effectiveness of the interventions. Even despite the relatively large numbers of patients reported [27,30]. No serious adverse events of the programmes have been reported by the included studies [26,28,30,31]. To our knowledge, there has been one further study by Miralpeix et al. evaluating a prehabilitation programme after our search was performed. This pilot study also demonstrated the feasibility and safety of a multimodal prehabilitation programme before interval cytoreductive surgery in ovarian cancer patients, with significant improvement of nutritional parameters [33].

Prehabilitation programmes have already been extensively assessed in other cancer sites. In colorectal cancer, results are promising, with significant reductions of complication rates up to 51% and a significantly shortened length of stay [20,21,34]. The majority of these programmes are multimodal, comprising exercise therapy, nutritional guidance, psychological interventions and treatment of intoxications and anaemia, with exercise being one of the most important mainstays [21,34]. A recent systematic review and meta-analysis comprising 3962 patients undergoing abdominal cancer surgery found significant reductions in complication rates and length of stay for unimodal (exercise or nutrition) and multimodal prehabilitation programmes [35]. In addition, a systematic review of prehabilitation in hepatobiliary, colorectal and upper gastrointestinal cancer surgery showed a reduced hospital stay of almost 2 days [17]. Similar results have been reported for bladder cancer, assessing both unimodal and multimodal prehabilitation programmes, with improvements found in complication rates, functional capacity and strength and quality of life [36,37,38,39].

The studies described above demonstrate the feasibility of introducing a prehabilitation programme in the preoperative period of gynaecological cancer patients, a patient group who may particularly benefit from prehabilitation. This cohort of women is characterised by modifiable risk factors that both predispose them to malignancy and increase the risk of postoperative morbidity and prolonged hospital stay [5,40,41,42]. Most patients with endometrial and ovarian cancer are overweight or obese, with the majority engaging in sedentary lifestyles, failing to meet national recommendations for exercise and nutrition [7,43,44]. Consequently, these women have a high burden of obesity-related comorbidities, particularly cardiovascular disease [45,46,47]. Although not consistently reported, study populations in this review were characterised by the presence of moderate comorbidities and high BMI. Malnourishment is also a common problem among gynaecological cancer patients, with reported rates up to 67% of patients [6,41,48]. Finally, cancer diagnosis confers significant psychological distress to patients, with additional stressors of infertility, female organ removal and induced menopause in this patient group. The prevalence of anxiety and depression amongst gynaecological cancer patients has subsequently been reported up to 30% [49].

This systematic review is the most recent and comprehensive literature search to date evaluating prehabilitation for gynaecological cancer patients. The majority of studies in this review evaluated a combination of cancer types, of which ovarian, (recurrent) endometrial and cervical cancer were the most prevalent. The results of the review are, however, limited by the nonrandomised and retrospective design of studies and small study populations. There was often a difference in baseline characteristics between groups, and several studies included benign conditions and other cancer types, introducing further bias [25,27,28,29,30]. Arnaboldi et al. assessed patients undergoing exenterative surgery, which is not standard primary treatment, prohibiting extrapolation of results to the general endometrial and cervical cancer population [25]. The variation in components of the programmes and the lack of detailed description of some interventions are also important limitations. In addition, differences in assessment of postoperative complications, quality of life, coping and cognitive functioning limit comparability of studies. Future studies should take this into account by including detailed descriptions of interventions and selecting outcome measures allowing comparability with other studies. Lastly, the study by Diaz-Feijoo et al. was included as a preliminary report prior to their later publication in 2022. After comparison with their published paper, we conclude no significant differences in methodology nor reported results [28,32].

Despite demonstrating the feasibility of prehabilitation, defining the essential components and attributes of a prehabilitation programme still poses a challenge. The exercise programmes assessed in this review generally included aerobic and resistance training, although not described in detail by all studies. This is in concurrence with the American College of Sports Medicine (ACSM) guidelines, recommending aerobic and resistance for cancer patients. However, the ACSM was unable to give recommendations for gynaecological cancer patients specifically due to limited data [50]. Following the variety of health impairments prevalent among gynaecological cancer patients, a prehabilitation programme should include both exercise and nutritional interventions, ideally in combination with psychological support and smoking cessation. Factors known to improve adherence and compliance include setting attainable goals and individualised guidance [51]. Possible strategies may include supervised individual or group exercise sessions and coaching sessions. Another challenge may be the limited time frame for prehabilitation in cancer patients, as it is dictated by the national and international recommendations on time between diagnosis and treatment [52,53]. Studies in this review implemented a programme varying from 5 days to over 6 months, with the multimodal programmes lasting around 2–3 weeks.

Prehabilitation may also provide an opportunity to introduce sustainable lifestyle changes, as current counselling on a healthy lifestyle is inadequate and incongruent with patients’ needs [54]. Cancer patients are in a ‘’teachable moment’’, willingly to modify their lifestyle in hope of achieving better health [55]. Currently, endometrial cancer patients are still more likely to die of cardiovascular disease than endometrial cancer [56]. Sustainable lifestyle changes could positively influence long-term survival and recurrence of cancer patients [57,58]. Therefore, it is clear that there is a need for future prospective randomised studies to assess the short-term and long-term impact of prehabilitation for gynaecological cancer patients.

## 5. Conclusions

The results of this systematic review support the view that prehabilitation is feasible within patients with gynaecological cancer. Although further research is needed to further delineate the clear benefits and essential components of a prehabilitation programme in this specific group, preliminary studies have already shown potential benefits in terms of length of hospital stay, weight loss and decreasing time to adjuvant chemotherapy. In combination with the extrapolated outcomes from similar treatments in other cancers, this sets down a clear need for investment in future prospective randomised studies, preferably with carefully controlled outcome measures designed to assess the impact and benefits for both the patient populations and health systems alike.

## Figures and Tables

**Table 1:** Studies on prehabilitation for ovarian cancer.

Study	Country Year	Study Design	Population (n) Age (years)	Cancer Type	Type of Programme and Duration	Outcome Measures	Outcomes
**Diaz-Feijoo [28]**	Spain, 2021	Prospective vs. retrospective cohort	n = 34 IG: 15 CG: 19 Age (median): IG: 55 CG: 60	Ovarian: 34	Physical therapy Nutrition Psychological therapy 2 weeks or more	Postoperative complications (Clavien–Dindo) Hospital stay Time until chemotherapy Compliance	Hospital stay: 5 vs. 7 days (IG vs. CG) *. Time until chemotherapy: 25 vs. 35 days (IG vs. CG) *. ICU stay NS Complications: NS Compliance: 80% No adverse events
**Fernández-Candela [30]**	Spain, 2021	Retrospective cohort	n = 107 IG: 48 CG: 59 Age (median): 60	Ovarian: 53 Colorectal: 32 Pseudomyxoma: 13 Others: 9	Nutrition (Atempero ^R^) 7 days	Postoperative complications (Clavien–Dindo) Hospital stay CRP Adherence	More visceral resections in IG * (>2 in 73% IG vs. 27% CG) Less HIPEC given in IG * Complications: NS Immunonutrition is independent protective factor for postoperative complications * (OR 0.247; 95%CI 0.071–0.859) Overall complications: NS Hospital stay: NS CRP: NS No adverse events
**Hertlein [26]**	Germany, 2018	Prospective cohort	n = 47 IG: 28 CG: 19 Age (median): IG: 70 CG: 68	Ovarian: 47	Nutrition (Impact ^R^) 10 days (5 preoperative)	Postoperative complications Length of stay Compliance	Postoperative complications: NS Length of stay: NS Preoperative compliance: 78.6% Postoperative compliance: 28.5% No adverse events
**Seibaek [27]**	Denmark, 2012	Prospective cohort	n = 145 IG: 55 CG: 90 Age (mean): IG: 63 CG: 58	Ovarian: 109 Benign: 36	Physical therapy Nutrition Psychological therapy Smoking cessation Duration: unknown	Quality of life (SF-36) Coping (SOC)	Higher physical functioning: CG vs. IG (8.58 points) * Other quality of life outcomes: NS Coping: NS

*: significant; CG: control group; IG: intervention group; NS: not significant; SF-36: Short-Form 36; SOC: sense of coherence.

**Table 2:** Studies on prehabilitation for endometrial and cervical cancer.

Study	Country Year	Study Design	Population (n) Age (years)	Cancer Type	Type of Programme and Duration	Outcome Measures	Outcomes
**Arnaboldi [25]**	Italy, 2015	Prospective cohort	n = 49 IG: 17 CG: 32 Age (median): 54	Cervix: 29 Endometrial: 7 Peritoneal: 1 Fallopian: 3 Vagina: 7 Vulva: 2	Psychological therapy Duration: unknown	Postoperative pain (VAS) Hospital stay	Postoperative pain: NS Hospital stay: NS
**Aubrey [29]**	Canada, 2021	Retrospective cohort	n = 48 IG: A: n = 39 B: n = 3 C: n = 6 Age (mean): 58	Endometrial: 31 Hyperplasia: 7 Adnexal mass: 7 Other: 4	Nutrition 12 weeks to 6 months	Weight loss Surgical time Blood loss Conversion Hospital stay	Mean weight loss 12.0 kg (9.7%) * Other surgical outcomes: NS Hospital stay: NS
**Carli [31]**	Canada, 2012	Case report	n = 1 Age: 88	Endometrial	Physical therapy Nutrition 3 weeks	Exercise tolerance (6 MWT) Cognitive function (RBANS) Quality of life (SF-36) Perioperative complications	Increased 6 MWT (91.2 m to 144.8 m) Cognitive function: RBANS increased from 58 (<1st percentile) to 81 (10 percentile) Improvements in both mental and physical components of SF-36 No complications No adverse events

*; significant; CG: control group; IG: intervention group; NS: not significant; RBANS: Repeatable Battery for the Assessment of Neuropsychological Status; VAS: Visual Analogue Scale; SF-36: Short-Form 36; 6 MWT: 6 minute walk test.

**Table 3:** Risk of bias analysis (ROBINS-I).

	Confounding	Selection of Participants	Classification of Interventions	Deviations from Intended Interventions	Missing Data	Measurement of Outcomes	Selection of Reported Results
**Arnaboldi [25]**	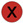	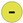	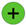	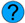	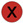	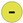	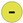
**Aubrey [29]**	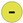	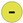	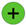	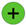	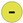	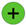	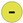
**Diaz-Feijoo [28]**	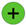	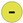	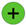	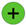	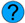	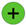	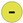
**Fernández-Candea [30]**	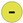	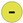	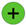	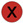	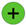	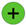	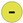
**Hertlein [26]**	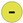	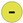	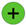	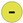	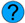	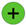	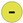
**Seibaek [27]**	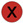	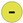	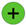	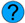	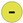	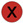	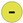

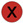
 Serious risk; 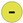 Moderate risk; 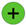 Low risk; 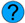 No information.

## Appendix A. Search Strategy

1 (“Genital neoplasms, female”[Mesh] OR Gynecological cancer*[tiab] OR gynecological oncology[tiab] OR Gynaecological cancer*[tiab] OR gynaecological oncology[tiab] OR female cancer*[tiab] OR endometrial carcinoma*[tiab] OR endometrial cancer*[tiab] OR endometrial neoplasm*[tiab] OR endometrium carcinoma*[tiab] OR endometrium cancer*[tiab] OR endometrium neoplasm*[tiab] OR uterine carcinoma*[tiab] OR uterine cancer*[tiab] OR uterine neoplasm*[tiab] OR vaginal carcinoma*[tiab] OR vaginal cancer*[tiab] OR vaginal neoplasm*[tiab] OR vulva carcinoma*[tiab] OR vulva cancer*[tiab] OR vulva neoplasm*[tiab] OR cervical carcinoma*[tiab] OR cervical cancer*[tiab] OR cervical neoplasm*[tiab] OR cervix carcinoma*[tiab] OR cervix cancer*[tiab] OR cervix neoplasm*[tiab] OR ovarian carcinoma*[tiab] OR ovarian cancer*[tiab] OR ovarian neoplasm*[tiab])

2 (“surgery” [Subheading] OR “surgical procedures, operative”[Mesh] OR “gynecologic surgical procedures”[Mesh] OR Surg*[tiab] OR Resection[tiab] OR operation*[tiab] OR operative [tiab] OR debulking [tiab] OR cytoreduct* [tiab] OR hysterectom* [tiab])

3 (“Preoperative Period”[Mesh] OR “Preoperative Care”[Mesh] OR “Preoperative Exercise”[Mesh] OR Preoperati*[tiab] OR Pre Operati*[tiab] OR Pre-operati*[tiab] OR Before Operati*[tiab] OR Before Procedure*[tiab] OR Before Surg*[tiab])

4 “Physical Fitness”[Mesh] OR “Physical Therapy Modalities”[MesH] OR “Physical Endurance”[Mesh] OR “Exercise”[MeSH] OR “Exercise Test”[Mesh] OR “Fitness Trackers”[Mesh] OR “Physical Exertion”[Mesh] OR “Exercise Therapy”[Mesh] OR “Sports”[Mesh] OR “Preoperative exercise”[Mesh] OR Physical fitness[tiab] OR Calisthenic*[tiab] OR Enduranc*[tiab] OR Exercis*[tiab] OR Fitness*[tiab] OR Training*[tiab] OR Gymnastic*[tiab] OR Multimodal Therap*[tiab] OR Multi-modal Therap*[tiab] OR Physical*[tiab] OR Physiotherap*[tiab] OR Prehabilitation[tiab] OR Pre-habilitation[tiab] OR Stamina[tiab]

OR “Diet, Food, and Nutrition”[Mesh] OR “nutrition therapy”[Mesh] OR “dietary supplements”[Mesh] OR Diet[tiab] OR Diets[tiab] OR dietary[tiab] OR Food*[tiab] OR Nutrition*[tiab] OR Nutrient*[tiab] OR Nutraceutical*[tiab] OR Neutraceutical*[tiab] OR Protein*[tiab] OR Proteins[tiab] OR Macronutrient*[tiab]

OR “Adaptation, Psychological”[Mesh] OR “Mental Health”[Mesh] OR “Psychology”[Mesh] OR “Psychology”[subheading] OR “psychotherapy”[Mesh] OR “psychosocial intervention”[Mesh] OR Psycholog*[tiab] OR Psychosocial*[tiab] OR Coping*[tiab] OR Acceptance*[tiab] OR Mental*[tiab] OR Mental support[tiab] OR neuropsychological[tiab] OR emotional adjustment[tiab]

OR “Smoking”[Mesh] OR “Smoking Cessation”[Mesh] OR “Smoking Cessation Agents”[Mesh] OR “Tobacco Use Cessation”[Mesh] OR “smoking reduction”[Mesh] OR “Tobacco Use Cessation Devices”[Mesh] OR Smok*[tiab] OR Tobacco[tiab]

OR “Alcoholic beverages”[Mesh:noexp] OR “alcohol drinking”[Mesh] OR “Alcohol abstinence”[Mesh] OR “binge drinking”[Mesh] OR Alcohol[tiab] OR drinking[tiab] OR alcoholic

5 (“Mortality”[Mesh] OR “mortality” [Subheading] OR “Death”[Mesh] OR “Morbidity”[Mesh] OR “wound healing”[Mesh] OR “surgical wound dehiscence”[Mesh] OR “length of stay”[Mesh] OR “Infections”[Mesh] OR “sepsis”[mesh] OR “pneumonia”[mesh] OR “urinary tract infections”[mesh] OR “pulmonary atelectasis”[mesh] OR “quality of life”[mesh] OR “shock”[mesh] OR “hemorrhage”[Mesh] OR “postoperative hemorrhage”[Mesh] OR “venous thrombosis”[Mesh] OR “pulmonary embolism”[mesh] OR “venous thromboembolism”[mesh] OR “pain”[mesh] OR Mortality[tiab] OR Death[tiab] OR Morbidity[tiab] OR wound healing[tiab] OR surgical wound dehiscence[tiab] OR length of stay[tiab] OR Infection*[tiab] OR sepsis[tiab] OR pneumonia[tiab] OR urinary tract infection*[tiab] OR pulmonary atelectasis[tiab] OR quality of life[tiab] OR QoL[tiab] OR HRQoL[tiab] OR shock[tiab] OR hemorrhage*[tiab] OR postoperative hemorrhage[tiab] OR venous thrombosis[tiab] OR pulmonary embolism[tiab] OR venous thromboembolism[tiab] OR pain[tiab] OR UTI[tiab] OR DVT[tiab] OR deep vein thrombosis[tiab] OR VTE[tiab] OR anesthesia reaction* [tiab] OR anaesthesia reaction*[tiab] OR complication*[tiab])

6 #1 AND #2 AND #3 AND #4 AND #5
